# Oligo/Amenorrhea Is an Independent Risk Factor Associated With Low Ovarian Response

**DOI:** 10.3389/fendo.2021.612042

**Published:** 2021-06-09

**Authors:** Kai-Lun Hu, Kwanghann Gan, Yue Ying, Junyan Zheng, Ruixue Chen, Jinglei Xue, Yiqing Wu, Yifeng Liu, Yimin Zhu, Lanfeng Xing, Dan Zhang

**Affiliations:** Key Laboratory of Reproductive Genetics (Ministry of Education) and Department of Reproductive Endocrinology, Women’s Hospital, School of Medicine, Zhejiang University, Hangzhou, China

**Keywords:** anti-müllerian hormone, menstrual cycle, oligomenorrhea, polycystic ovary syndrome, ovarian response

## Abstract

**Capsule:**

Oligo/amenorrhea is an independent risk factor of low ovarian response but not high ovarian response, particularly in women with low AMH levels.

**Objective:**

To investigate the association of menstrual cycle length (MCL) with anti-Müllerian hormone (AMH) and ovarian response.

**Methods:**

This was a retrospective cohort study. A total of 7471 women who underwent ovarian stimulation and oocyte retrieval were enrolled. The main outcome was the number of oocytes retrieved.

**Main Results:**

A total of 5734 patients were eligible for analysis. In women without polycystic ovary syndrome (PCOS), serum AMH levels and antral follicle count were significantly lower in women with short cycles and higher in women with oligo/amenorrhea than those with a normal menstrual cycle. In women with PCOS, compared to women with a normal menstrual cycle, women with short cycles and women with oligo/amenorrhea showed higher antral follicle count and higher serum AMH levels. Compared with the 0-25th range group of AMH levels, 75-100th percentile groups showed a significantly increased rate of oligo/amenorrhea in women with and without PCOS [adjusted odds ratio (OR) =1.9 (1.04, 3.46), 2.4 (1.70, 3.35)]. In women without PCOS, the low ovarian response was more common in women with short cycles and less common in women with oligo/amenorrhea compared to women with normal cycles [OR=3.0 (2.38, 3.78), 0.7 (0.55, 0.96), respectively]. When adjusted for AMH levels, both short cycles and oligo/amenorrhea were associated with an increased risk of low response [adjusted OR=1.3 (1.02, 1.75), 1.3 (0.93, 1.86), respectively]. In women without PCOS and with low AMH levels, the low ovarian response was more common in women with short cycles as well as in women with oligo/amenorrhea [OR=1.5 (1.08, 1.98), 1.7 (1.08, 2.69), adjusted OR=1.2 (0.86, 1.74), 2.2 (1.31, 3.82), respectively].

**Conclusion:**

AMH levels are significantly associated with increased risk of oligo/amenorrhea in women with and without PCOS. AMH is an indispensable confounder in the association between MCL and ovarian response in women without PCOS. Oligo/amenorrhea is an independent risk factor associated with a low ovarian response in women without PCOS, particularly those with low AMH levels.

## Introduction

Infertility is defined as the inability of a couple to get pregnant after one year of regular unprotected intercourse (1). Infertility is also a prevalent medical condition affecting 10% to 20% of couples across different countries (2, 3). *In vitro* fertilization and embryo transfer (IVF-ET) has allowed millions of infertile couples worldwide to successfully conceive since 1978 (4). Gonadotropins are regularly used to stimulate ovarian follicle growth in IVF procedures, and ovarian response to gonadotropins can largely influence the chance of success in an IVF cycle (5). A good marker to predict ovarian response may help clinicians to manage the doses of gonadotropins and other procedures in an IVF cycle.

In females, serum AMH arises mainly from pre-antral and small antral follicles (6, 7), and serum AMH levels are proportional to the number of growing follicles in the ovaries (6, 8). Many studies indicate that serum AMH levels can be used to predict IVF cycle success (9–11). Currently, serum AMH levels are considered as the best available measure of ovarian reserve under a variety of clinical conditions, including infertility treatment, the forecasting of reproductive lifespan, ovarian surgery, and gonadotoxic cancer therapy (12–14). Additionally, emerging evidence shows that serum AMH levels can be used to predict ovarian response to exogenous gonadotropins (7, 14–16).

Menstruation is cyclic in response to the interactions of hormones secreted from the hypothalamus, pituitary, and ovaries. The menstrual cycle length (MCL) is the time frame from the first day of menstrual bleeding of one cycle to the onset of menses of the next cycle. The majority of MCL is between 25 to 35 days in women (17). MCL can be affected by several diseases, such as polycystic ovarian syndrome (PCOS), endometriosis, hyperprolactinemia, gynecological cancer, etc. Recent studies indicate that serum AMH levels were higher in PCOS women with menstrual disturbance when compared to PCOS women with regular cycles (18). Serum AMH levels are positively associated with MCL in healthy women, which puts forth a new notion of utilizing MCL to predict possible AMH associated outcomes, including ovarian response (19–21). We hypothesize that the various MCL in women may reflect some AMH-related clinical characteristics. The primary aim of our study is to explore the association of MCL with AMH and ovarian response in women undergoing ovarian stimulation.

## Materials and Methods

This study was approved by the Ethics Committee of Women’s Hospital of Zhejiang University (reference: IRB-20200016-R) and written informed consent was obtained from each participant. Patients undergoing their first ovarian stimulation cycle from June 2017 to September 2019 in Women’s Hospital of Zhejiang University were enrolled in this study. Patients were excluded if they had a history of oophorectomy, or if they had been previously diagnosed with endometriosis or hyperprolactinemia or any other reasons except polycystic ovarian syndrome (PCOS) that might cause irregular menstrual cycle, or if their menstrual cycle was maintained by drugs, or if they were not measured with serum AMH levels, or if their MCL data, or oocyte retrieval data were not clearly recorded in the dataset. In most cases, the most commonly reported MCL in the year preceding the treatment cycle was recorded in the dataset. The average MCL was used in several women who had different lengths of the cycle (for example, 28 days in this cycle and 29 days in the next cycle). Cycles with MCL < 26 days were defined as short cycles and MCL > 35 days was defined as oligo/amenorrhea according to previous studies (18, 22, 23). A cycle length of 26-35 days was considered as a normal cycle in this study.

Ovarian stimulation was induced either in the long or short GnRH agonist or GnRH antagonist protocol. In the long protocol, GnRH agonist was administered in the mid-luteal phase of the cycle preceding ovarian stimulation. In the short protocol, the GnRH agonist was administered on day 2 of the ovarian stimulation cycle. In the GnRH antagonist protocol, GnRH antagonist was administered according to the follicle development and serum estradiol levels (around 5 or 6 days after Gn treatment). Recombinant follicle stimulation hormone (FSH) (Gonafen, Pricon) and/or human menopausal gonadotrophin (Livzon, China) was commenced on day 2 of the cycle at a doses of 75-225 IU, and the doses were adjusted according to the ovarian response (follicle count under ultrasound and/or serum E2 levels). When the size of at least two leading follicles reached 18 mm, ovulation was triggered by the administration of recombinant HCG (6500 IU; Livzon, China); then, the oocyte was retrieved 36–38 h later. Low, moderate, and high ovarian response in this study were defined as oocyte retrieval number ≤ 3, 3-15, and ≥16, respectively, according to a previous study (24).

Serum AMH, FSH, luteinizing hormone (LH), progesterone, and estradiol levels were measured by electrochemiluminescence on menstrual cycle day 2 or day 3 within 12 months before the treatment cycle (Sandhofer Strasse 116,68305 Mannheim, Germany). The assay kit for AMH measurement could detect a range from 0.01 to 23 ng/ml. In some cases (less than ten) that the test value would beyond this range, we chose the value of 0.01 or 23 ng/ml for substitution (0.01 ng/ml for “< 0.01 ng/ml” and 23 ng/ml for “> 23 ng/ml”). The intra- and inter-assay coefficients of variation were all less than 10%.

Comparison between groups was performed using the independent sample t-test, ANOVA test, Chi-square test (*χ* ^2^), and non-parametric test as appropriate. Logistic regression analysis was used to determine the risk of factors and the results were expressed with the odds ratio (OR) or adjusted OR (aOR) with 95% confidence intervals (CI). All statistical procedures were run on SPSS version 22.0. P < 0.05 was considered a statistically significant difference.

## Results

### Characteristics of Women With Short Cycles, Normal MCL and Oligo/Amenorrhea

**Figure 1:**
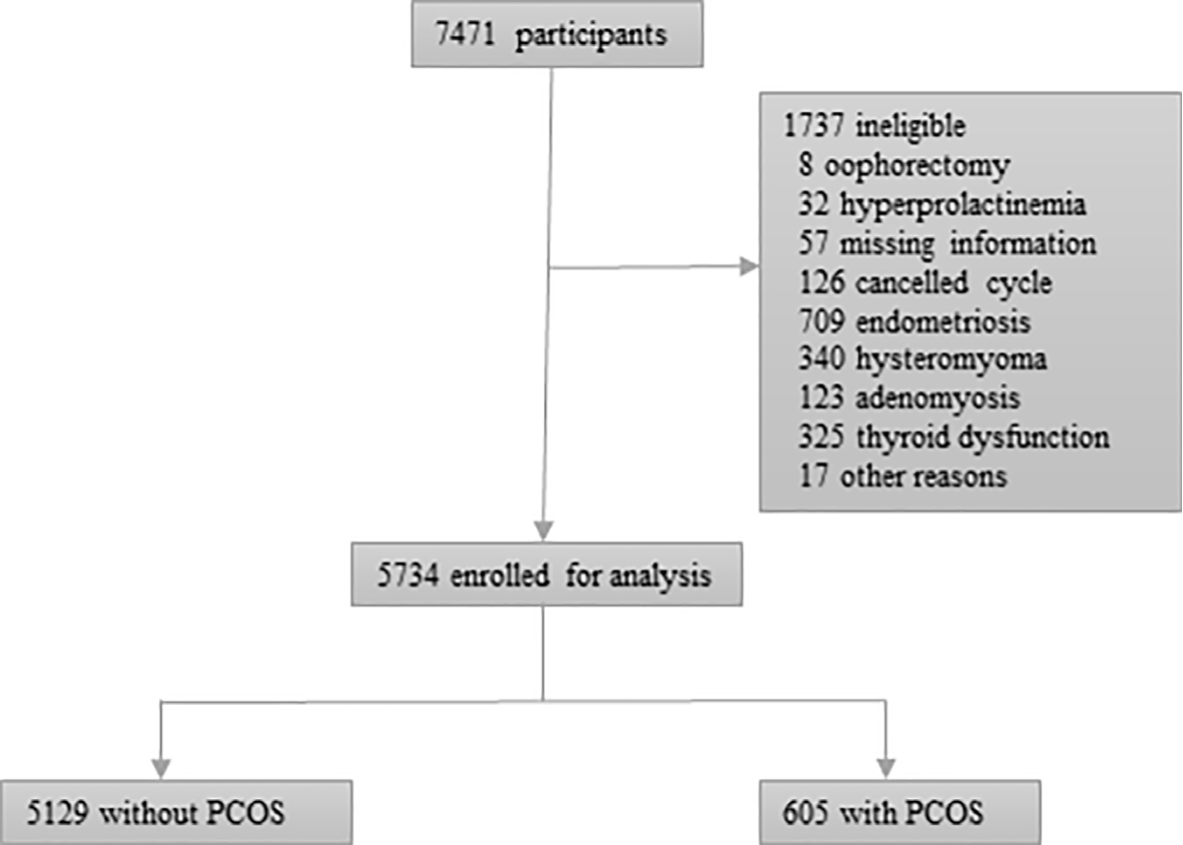
Flowchart for eligible participants.

7471 women met the inclusion criteria and 5734 women were eligible for analysis. The reason for excluded women could be seen in **Figure 1**. 5129 women did not meet the diagnosing criteria of PCOS and 605 women had a diagnosis of PCOS.

In women without PCOS, compared to women with a cycle length of 26-35 days, women with short cycles had higher average age, a longer infertility time, a higher rate of gravidity and parity, and a shorter mean bleeding duration. Women with oligo/amenorrhea showed a lower average age, a higher average BMI, a longer infertility time, a lower rate of gravidity and parity, and a longer mean bleeding duration (**Table 1**). Additionally, women with short cycles showed higher basal FSH levels and lower LH levels, whereas women with oligo/amenorrhea showed lower basal FSH levels and higher LH levels. Serum AMH levels, antral follicle count, and oocyte retrieval numbers were significantly lower in women with short cycles and higher in women with oligo/amenorrhea than those with a normal menstrual cycle. The total gonadotropin doses were significantly lower in women with oligo/amenorrhea (**Table 1**). The *P* value for each parameter comparison could be seen in **Table 1**.

**Table 1:** Characteristics of women without a diagnosis of PCOS (n=5129).

Menstrual cycle length (days)	<26 (n = 367)	26-35 (n = 4205)	>35 (n = 557)	*P* value^1^
Age (y)	33.3 ± 5.2	31.6 ± 4.7	30.7 ± 4.3	<0.00001
BMI (kg/m2)	21.6 ± 2.4	21.6 ± 2.6	22.0 ± 2.8	<0.01
Menarche age (y)	14.0 ± 1.4	13.9 ± 1.4	14.0 ± 1.5	NS
Infertility years (y)	3.7 ± 3.3	3.3 ± 2. 9	3.7 ± 3.0	<0.01
Bleeding duration (days)	5.6 ± 1.5	5.8 ± 1.4	6.1 ± 1.3	<0.00001
MCL (days)	24 (25, 25)	30 (28, 30)	38 (40, 45)	<0.00001
Gravidity				<0.00001
0	139 (38%)	1822 (43%)	281 (50%)	
≥1	228 (62%)	2383 (57%)	276 (50%)	
Parity				<0.00001
0	257 (70%)	3352 (80%)	468 (84%)	
≥1	110 (30%)	853 (20%)	89 (16%)	
Antral follicle count	9.4 ± 5.1	12.3 ± 5.2	13.8 ± 5.4	<0.00001
Basal hormone levels^2^				
FSH mIU/ml	8.7 ± 4.1	7.2 ± 3.1	6.4 ± 2.3	<0.00001
Estradiol pmol/l	147 ± 98	118 ± 68	120 ± 89	NS
Progesterone nmol/l	1.1(0.7,1.5)	1.1 (0.8,1.5)	1.0 (0.7,1.4)	NS
LH mIU/ml	4.7 ± 2.9	5.0 ± 2.4	6.0 ± 3.8	<0.00001
AMH ng/ml	1.7 ± 1.6	2.9 ± 2.1	4.2 ± 2.8	<0.00001
Testosterone nmol/l	0.7(0.4,1.0)	0.7 (0.5,1.0)	0.8 (0.6,1.1)	NS
Gonadotropin dose (IU)	1947 ± 826	2037 ± 747	1931 ± 718	<0.00001
Oocyte retrieval number	6.8 ± 5.4	10.4 ± 6.6	11.8 ± 7.1	<0.00001

NS, not significant (P > 0.05). Mean ± SD or median (IQR) as appropriate.

^1^χ^2^, non-parametric test, or t test as appropriate.

^2^Day 2 or day 3 of the menstrual cycle.

In women with PCOS, compared to women with a cycle length of 26-35 days, women with short cycles and women with oligo/amenorrhea showed higher antral follicle count and higher serum AMH levels (**Supplemental Table 1**). Age, BMI, infertile age, bleeding duration, parity, gravidity, FSH levels, and LH levels were not statistically significant between the three groups. The parameter comparisons could be seen in **Supplemental Table 1**.

### Association Between Serum AMH Levels and MCL

To investigate whether elevated AMH levels were associated with oligo/amenorrhea, we stratified AMH levels by 0-25th, 25-50th, 50-75th, 75-100th percentile.

The range of AMH values in women without PCOS were min-1.33, 1.34-2.47, 2.48-4.06, and 4.07-max, respectively. Compared with the 0-25th range group, 75-100th percentile groups showed a significantly increased rate of oligo/amenorrhea [crude OR = 3.9 (2.99, 5.05), adjusted OR = 2.4 (1.70, 3.35)] (**Table 2**).

**Table 2:** AMH breakdown in women with and without PCOS.

Menstrual cycle length	<26	26-35	>35	OR^1^ value (95%CI)	Adjusted^2^ OR value (95%CI)
**Women without PCOS (n=5129)**					
AMH, ng/mL	1.7 ± 1.6	2.9 ± 2.1	4.2 ± 2.8		
Distribution/value					
min-1.33	199 (54%)	1006 (25%)	81 (15%)	Reference	Reference
1.34-2.47	95 (26%)	1103 (26%)	86 (15%)	1.1 (0.78, 1.46)	0.9 (0.69, 1.21)
2.48-4.06	44 (12%)	1110 (26%)	125 (22%)	1.6 (1.21, 2.16)	1.2 (0.83, 1.63)
4.07-max	29 (8%)	986 (23%)	265 (48%)	3.9 (2.99, 5.05)	2.4 (1.70, 3.35)
**Women with PCOS (n=605)**					
AMH, ng/mL	8.9 ± 6.8	6.9 ± 4.4	8.1 ± 4.6		
Distribution/value				Reference	Reference
min-4.43	4 (31%)	54 (36%)	93 (21%)		
4.44-7.09	1 (7%)	32 (21%)	119 (27%)	2.2 (1.36, 3.73)	2.1 (1.23, 3.66)
7.10-10.08	4 (31%)	34 (23%)	113 (26%)	1.9 (1.13, 3.04)	1.7 (1.00, 2.97)
10.09-max	4 (31%)	31 (21%)	116 (26%)	2.1 (1.25, 3.41)	1.9 (1.04, 3.46)

^1^Odds ratio for oligo/amenorrhea.

^2^Adjusted for female age, infertile age, BMI, FSH levels, LH levels, gravidity, parity, antral follicle count.

The range of AMH values in women with PCOS were min-4.43, 4.44-7.09, 7.10-10.08, and 10.09-max, respectively. Compared with the 0-25th range group, 25-50th, 50-70th, 75-100th percentile groups showed a significantly increased rate of oligo/amenorrhea [adjusted OR = 2.1 (1.23, 3.66), 1.7 (1.00, 2.97), 1.9 (1.04, 3.46), respectively] (**Table 2**).

### Characteristics of Patients With High, Moderate, and Low Ovarian Response

In the 5129 women without PCOS, there were 805 women with a low response, 3305 with a moderate response, and 1019 with a high response. Women with low response showed a higher average age and BMI, a lower MCL, a higher rate of gravidity and parity, higher serum FSH levels and estradiol levels, and lower AMH levels and antral follicle count. Other variables comparison could be seen in **Table 3**.

**Table 3:** Characteristics of women without PCOS and with the low, moderate, and high ovarian response (n=5129).

Ovarian response	Low (n = 805)	Moderate (n = 3305)	High (n = 1019)	*P* value^1^
Age (y)	34.8 ± 5.3	31.4 ± 4.3	29.4 ± 3.7	<0.00001
BMI (kg/m2)	22.0 ± 2.7	21.6 ± 2.6	21.3 ± 2.6	<0.0001
Menarche age (y)	13.9 ± 1.3	13.9 ± 1.4	13.9 ± 1.4	NS
Infertility years (y)	3.8 ± 3.6	3.3 ± 2.8	3.0 ± 2.4	<0.00001
Bleeding duration (days)	5.7 ± 1.4	5.8 ± 1.3	6.0 ± 1.5	<0.00001
Menstrual cycle	28 (27, 30)	30 (28, 30)	30 (28, 32)	<0.00001
Gravidity (≥1)	483 (60%)	1859 (56%)	545 (54%)	<0.05
Parity (≥1)	232 (29%)	676 (20%)	144 (14%)	<0.00001
Antral follicle count	6.8 ± 4.3	12.4 ± 4.6	16.0 ± 4.5	<0.00001
Basal hormone levels^2^				
FSH mIU/ml	9.7 ± 5.4	6.9 ± 2.2	6.0 ± 1.5	<0.00001
Estradiol pmol/l	135 ± 100	120 ± 68	112 ± 58	<0.00001
Progesterone nmol/l	1.1(0.7,1.5)	1.1(0.8,1.5)	1.1 (0.8,1.4)	NS
LH mIU/ml	5.0 ± 3.6	4.9 ± 2.3	5.6 ± 2.7	<0.00001
AMH ng/ml	1.0 ± 1.0	2.8 ± 1.9	5.0 ± 2.5	<0.00001
Testosterone nmol/l	0.7 (0.4,1.0)	0.7 (0.5,1.0)	0.8 (0.5,1.1)	NS
Gonadotropin dose (IU)	1592 ± 894	2139 ± 705	1970 ± 637	<0.00001
GnRH antagonist	258 (32%)	1534 (47%)	353 (35%)	<0.00001

NS, not significant (P > 0.05). Mean ± SD or median (IQR) as appropriate.

^1^χ^2^ or ANOVA test as appropriate.

^2^Day 2 or day 3 of the menstrual cycle.

In the 605 women without PCOS, there were 35 women with a low response, 293 with a moderate response, and 277 with a high response. Women with low response showed a higher average age, higher serum FSH levels, lower AMH levels, and a lower antral follicle count. Other variables comparison could be seen in **Supplemental Table 2**.

### Association Between MCL and Ovarian Response

In women without PCOS, the low ovarian response was more common in women with short cycles and less common in women with oligo/amenorrhea compared to women with normal cycles [OR=3.0 (2.38, 3.78), 0.7 (0.55, 0.96), respectively]. When adjusted for AMH levels, both short cycles and oligo/amenorrhea were associated with an increased risk of low response [adjusted OR=1.3 (1.02, 1.75), 1.3 (0.93, 1.86), respectively]. The result was similar when adjusted for AMH levels and other potential confounders [adjusted OR=1.2 (0.87, 1.55), 1.5 (1.05, 2.22), respectively] (**Table 4**).

**Table 4:** The association of oligo/amenorrhea with low ovarian response in women with and without PCOS.

Ovarian response	High/moderate	Low	OR value (95%CI)	Adjusted OR value (95%CI)
Women without PCOS (n=5129)				
26-35	3587 (85%)	618 (15%)	Reference	Reference
<26	242 (66%)	125 (34%)	3.0 (2.38, 3.78)	1.3 (1.02, 1.75)^1^ 1.2 (0.87, 1.55)^2^
>35	495 (89%)	62 (11%)	0.7 (0.55, 0.96)	1.3 (0.93, 1.86)^1^ 1.5 (1.05, 2.22)^2^
Women without PCOS with low AMH^3^ levels (n=1286)				
26-35	555 (55%)	451 (45%)	Reference	Reference
<26	91 (46%)	108 (52%)	1.5 (1.08, 1.98)	1.2 (0.86, 1.74)^2^
>35	34 (42%)	47 (58%)	1.7 (1.08, 2.69)	2.2 (1.31, 3.82)^2^
Women with PCOS (n=605)				
26-35	140 (93%)	11 (7%)	Reference	Reference
<26	12 (92%)	1 (8%)	1.1 (0.13, 8.93)	1.2 (0.14, 10.51)^1^ 1.0 (0.09, 11.01)^2^
>35	148 (95%)	23 (5%)	0.7 (0.33, 1.47)	0.8 (0.38, 1.74)^1^ 0.9 (0.38, 2.05)^2^

^1^Adjusted for serum AMH levels.

^2^Adjusted for female age, BMI, infertile age, parity, gravidity, FSH levels, LH levels, estradiol levels, AMH levels, antral follicle number.

^3^AMH levels below 1.34 ng/ml.

In women without PCOS and with low AMH levels, the low ovarian response was more common in women with short cycles as well as in women with oligo/amenorrhea compared to women with normal cycles [OR=1.5 (1.08, 1.98), 1.7 (1.08, 2.69), adjusted OR=1.2 (0.86, 1.74), 2.2 (1.31, 3.82), respectively] (**Table 4**).

In women with PCOS, there was no statistically significant difference of the low ovarian response rate in women with short cycles, normal cycles, and oligo/amenorrhea (**Table 4**).

## Discussion

In the present study, we show that elevated AMH levels are significantly associated with increased risk of oligo/amenorrhea in women with and without PCOS. AMH is an indispensable confounder in the association between MCL and ovarian in women without PCOSresponse. Oligo/amenorrhea is an independent risk factor associated with a low ovarian response, particularly in women with low AMH levels.

AMH is predominantly known for its roles in the ovary. Recent studies have uncovered the neuroendocrine functions of AMH in the hypothalamus and pituitary (25). AMHR2 is broadly expressed in various brain area and cell types involved in central control of reproduction, including the hypothalamic gonadotropin-releasing hormone (GnRH) neurons (26). Peripheral administration of AMH can stimulate GnRH secretion and subsequent LH secretion to the levels equivalent to generate an ovulatory surge (26). Additionally, AMH directly and specifically regulates female FSH secretion in the pituitary (27). These studies indicate that AMH may participate in the interaction of hormones involved in the regulation of MCL. It is likely that elevated AMH levels may regulate the frequency of GnRH/LH pulse and affect the process of ovulation and menstrual cycle through the hypothalamus-pituitary-ovary axis (26, 28). Our result is consistent with previous findings that patients with higher AMH levels have longer MCL (18–21). MCL in women with the irregular menstrual cycle is gradually normalized with aging (29), but this has not been well explained. Our study shows that MCL is significantly associated with serum AMH levels when adjusted for potential confounding factors. Increased female age is significantly associated with a reduced risk of oligo/amenorrhea but it is insignificant in AMH adjusted model. It is likely that the gradually normalized MCL with aging can be explained by the progressively decreased circulating AMH levels (8).

The convenience of testing AMH any time throughout the menstrual cycle makes it a good marker for the prediction of ovarian reserve and ovarian response to gonadotropin stimulation (16). Considering that elevated serum AMH levels were significantly associated with an increased rate of oligo/amenorrhea, we hypothesize that MCL can be used to predict ovarian response. A previous retrospective observational study shows that the high value of MCL can increase the risk of ovarian hyperstimulation syndrome and MCL shortening is a risk factor of ovarian aging and poor ovarian response in normo-ovulatory infertile women (30). There are two limitations in this study for the least. The sample size is not large and potential confounders, including AMH and female age, are not adjusted (30). A recent meta-analysis also highlights that MCL shortening in women with regular menstrual cycles is associated with reduced ovarian response (31). Our study is consistent with these results showing short cycles is a risk factor for low ovarian response independent of age and AMH levels in women without PCOS. Interestingly, we find the low ovarian response is less common in women with oligo/amenorrhea and the trend can be reversed when adjusted for AMH levels in women without PCOS, indicating that AMH may be a key confounding factor when MCL is used to predict AMH associated outcomes. Then we are interested in whether oligo/amenorrhea is associated with low ovarian response in a certain range of AMH levels. We found oligo/amenorrhea is a risk factor in women without PCOS and with AMH levels in the 0-25th percentile but not in the 25-50th, 50-75th, 75-100th percentile (data not shown for 25-50th, 50-75th, 75-100th percentile). We define low ovarian response as oocyte retrieval number ≤ 3 in this study, and these women are unlikely to have a good pregnancy outcome (32). A recent study indicates that women with very low AMH values can have *in vitro* fertilization success (33). Our study shows that women with low AMH values are more likely to have very few oocytes collected if they show oligo/amenorrhea compared to those with the normal menstrual cycle. Therefore, it worths attention to the potential causes of oligo/amenorrhea in women undergoing ovarian stimulation and oocyte retrieval, particularly in women without PCOS and with low AMH levels. Probably due to the small number of women diagnosed with PCOS, we did not find oligo/amenorrhea is associated with low ovarian response in these women. Future studies investigating oligo/amenorrhea and outcomes in women diagnosed with PCOS will be interesting because oligo/amenorrhea is common in these women and it will provide good guidance for clinical practice.

The main limitation of this study is its retrospective study design. Although we adjusted our statistical analysis for a number of known and suspected confounders, some potential confounding factors may have an impact on the result. Oligo/amenorrhea can be caused by a variety of physiopathological factors, such as endometriosis, hyperprolactinemia, thyroid dysfunction, chemoradiotherapy, etc. Even we excluded potential conditions that may cause an irregular menstrual cycle, it is difficult to rule out all patients with at least one of these conditions. Furthermore, the recorded MCL of some patients in the dataset may not accurately be the real MCL in patients. For example, patients may report 2 months (60 days) if their true MCL is 58 days or 62 days. Previous studies indicate that cycles with lengths of 30 to 31 days have the highest fecundity, and conceptions after shorter and longer cycles are more likely to be spontaneously aborted (34). Additionally, Chinese women with menstrual cycle lengths >29 days are less likely to get pregnant (35). Because the pregnancy outcome in many of the enrolled women is not available, we did not analyze the association of MCL with pregnancy outcome in this study but it is promising for future investigations.

To summarize, our study shows that elevated AMH levels are significantly associated with increased risk of oligo/amenorrhea in women with and without PCOS. Serum AMH is an important confounder for MCL to predict ovarian response. Oligo/amenorrhea is an independent risk factor associated with the low ovarian response in women without PCOS, particularly those with low AMH levels. Future studies using MCL to predict other AMH associated outcomes are promising.

## Data Availability Statement

The original contributions presented in the study are included in the article/**Supplementary Material**. Further inquiries can be directed to the corresponding author.

## Ethics Statement

The studies involving human participants were reviewed and approved by the Ethics Committee of Women’s Hospital of Zhejiang University (reference: IRB-20200016-R). Written informed consent for participation was not required for this study in accordance with the national legislation and the institutional requirements.

## Author Contributions

K-LH reviewed the literature, designed the study, analyzed the data, wrote the manuscript and designed the tables. All authors participated in analysis or interpretation of data for the work. KG and DZ provided some key ideas for this manuscript. All authors contributed to the article and approved the submitted version.

## Funding

This study is supported by the National Key Research and Development Program of China (2018YFC1005003, 2017YFC1001003), the National Natural Science Foundation of China (No. 81974224, 81771535), the Fundamental Research Funds for the Central Universities, the Natural Science Foundation of Zhejiang Province (No. LZ18H040001), Zhejiang University Scholarship for Outstanding Doctoral Candidates, and Zhejiang University Education Foundation Global Partnership Fund.

## Conflict of Interest

The authors declare that the research was conducted in the absence of any commercial or financial relationships that could be construed as a potential conflict of interest.
