# Supplementary material for: Oligo/Amenorrhea Is an Independent Risk Factor Associated With Low Ovarian Response
Source: Front Endocrinol (Lausanne). 2021 Jun 9;12:612042. doi: 10.3389/fendo.2021.612042 (PMC8220146; doi:10.3389/fendo.2021.612042)
Supplement: Supplementary file 1 [file Table_1.docx]

Supplemental Table 1. Characteristics of women with a diagnosis of PCOS (n=605).

| Menstrual cycle length (days) | <26  (n=13) | 26-35  (n=151) | >35  (n=441) | *P* value^1^ |
| --- | --- | --- | --- | --- |
| Age (y) | 30.9±5.7 | 28.9±3.7 | 29.6±3.9 | 0.08 |
| BMI (kg/m2) | 21.8±2.6 | 22.8±3.0 | 22.5±2.7 | NS |
| Menarche age (y) | 14.0±0.7 | 13.7±1.3 | 13.9±1.4 | NS |
| Infertility years (y) | 2.7±1.6 | 3.3±2.2 | 3.4±2.4 | NS |
| Bleeding duration (days) | 6.2±0.8 | 6.3±1.4 | 6.4±1.3 | NS |
| MCL (days) | 24 (23, 25) | 30 (30, 33) | 53 (44, 72) | <0.00001 |
| Gravidity |  |  |  | NS |
| 0 | 8 (62%) | 104 (69%) | 269 (61%) |  |
| ≥1 | 5 (38%) | 47 (31%) | 172 (39%) |  |
| Parity |  |  |  | NS |
| 0 | 13 (100%) | 137 (91%) | 394 (89%) |  |
| ≥1 | 0 (0%) | 14 (9%) | 47 (11%) |  |
| Antral follicle count | 19.1±7.6 | 17.5±6.6 | 19.0±5.5 | 0.03 |
| Basal hormone levels^2^ |  |  |  |  |
| FSH mIU/ml | 7.4±3.2 | 6.0±1.8 | 6.0±2.1 | 0.09 |
| Estradiol pmol/l | 157±89 | 133±63 | 125±99 | NS |
| Progesterone nmol/l | 1.2(0.7,1.7) | 0.9(0.6,1.4) | 0.9(0.6,1.3) | NS |
| LH mIU/ml | 11.9±7.2 | 8.4±6.3 | 9.3±5.7 | 0.08 |
| AMH ng/ml | 8.9±6.8 | 6.9±4.4 | 8.1±4.6 | 0.01 |
| Testosterone nmol/l | 1.2(0.7,2.1) | 1.2(0.8,1.5) | 1.1(0.7,1.5) | NS |
| Gonadotropin dose (IU) | 1976±877 | 1979±763 | 1799±723 | 0.03 |
| Oocyte retrieval number | 16.1±11.2 | 15.6±10.8 | 16.2±9.0 | NS |

NS, not significant (*P*>0.05). Mean±SD or median (IQR) as appropriate

1. *χ*^2^, non-parametric test, or *t* test as appropriate.

2. Day 2 or day 3 of the menstrual cycle.
