# Supplementary material for: Oligo/Amenorrhea Is an Independent Risk Factor Associated With Low Ovarian Response
Source: Front Endocrinol (Lausanne). 2021 Jun 9;12:612042. doi: 10.3389/fendo.2021.612042 (PMC8220146; doi:10.3389/fendo.2021.612042)
Supplement: Supplementary file 2 [file Table_2.docx]

Supplemental Table 2. Characteristics of women with PCOS and with the low, moderate, and high ovarian response (n=605).

| Ovarian response | Low  (n=35) | Moderate  (n=293) | High  (n=277) | *P* value^1^ |
| --- | --- | --- | --- | --- |
| Age (y) | 31.9±5.7 | 29.8±3.8 | 28.7±3.6 | <0.00001 |
| BMI (kg/m2) | 23.6±3.3 | 22.6±2.8 | 22.4±2.6 | <0.05 |
| Menarche age (y) | 13.6±1.4 | 13.9±1.4 | 13.9±1.4 | NS |
| Infertility years (y) | 4.3±2.7 | 3.5±2.5 | 3.1±2.1 | <0.05 |
| Bleeding duration (days) | 6.6±1.5 | 6.3±1.4 | 6.4±1.3 | NS |
| Menstrual cycle | 38 (31, 60) | 45 (35, 60) | 45 (35, 60) | NS |
| Gravidity (≥1) | 13 (37%) | 107 (37%) | 104 (38%) | NS |
| Parity (≥1) | 7 (20%) | 29 (10%) | 25 (9%) | NS |
| Antral follicle count | 13.6±8.1 | 17.8±5.8 | 20.2±5.0 | <0.00001 |
| Basal hormone levels^2^ |  |  |  |  |
| FSH mIU/ml | 7.9±5.3 | 6.4±1.7 | 5.5±1.3 | <0.00001 |
| Estradiol pmol/l | 133±116 | 127±106 | 129±67 | NS |
| Progesterone nmol/l | 0.9(0.4,1.3) | 0.9(0.6,1.3) | 1.0(0.7,1.4) | NS |
| LH mIU/ml | 7.8±5.5 | 8.9±5.5 | 9.6±6.3 | NS |
| AMH ng/ml | 5.7±5.6 | 6.8±4.1 | 9.2±4.6 | <0.00001 |
| Testosterone nmol/l | 1.0(0.5,1.3) | 1.0(0.7,1.5) | 1.2(0.9,1.7) | NS |
| Gonadotropin dose (IU) | 1808±927 | 1923±749 | 1773±697 | 0.05 |
| GnRH antagonist | 15 (43%) | 152 (52%) | 158 (57%) | NS |

NS, not significant (*P*>0.05). Mean±SD or median (IQR) as appropriate

1. *χ*^2^ or ANOVA test as appropriate.

2. Day 2 or day 3 of the menstrual cycle.
